# Small Interfering RNA Targeting M2 Gene Induces Effective and Long Term Inhibition of Influenza A Virus Replication

**DOI:** 10.1371/journal.pone.0005671

**Published:** 2009-05-22

**Authors:** Hong-Yan Sui, Guang-Yu Zhao, Jian-Dong Huang, Dong-Yan Jin, Kwok-Yung Yuen, Bo-Jian Zheng

**Affiliations:** 1 Department of Microbiology, The University of Hong Kong, Pokfulam, Hong Kong SAR, China; 2 Department of Biochemistry, The University of Hong Kong, Pokfulam, Hong Kong SAR, China; Institute of Molecular and Cell Biology, Singapore

## Abstract

RNA interference (RNAi) provides a powerful new means to inhibit viral infection specifically. However, the selection of siRNA-resistant viruses is a major concern in the use of RNAi as antiviral therapeutics. In this study, we conducted a lentiviral vector with a H1-short hairpin RNA (shRNA) expression cassette to deliver small interfering RNAs (siRNAs) into mammalian cells. Using this vector that also expresses enhanced green fluorescence protein (EGFP) as surrogate marker, stable shRNA-expressing cell lines were successfully established and the inhibition efficiencies of rationally designed siRNAs targeting to conserved regions of influenza A virus genome were assessed. The results showed that a siRNA targeting influenza M2 gene (siM2) potently inhibited viral replication. The siM2 was not only effective for H1N1 virus but also for highly pathogenic avian influenza virus H5N1. In addition to its M2 inhibition, the siM2 also inhibited NP mRNA accumulation and protein expression. A long term inhibition effect of the siM2 was demonstrated and the emergence of siRNA-resistant mutants in influenza quasispecies was not observed. Taken together, our study suggested that M2 gene might be an optimal RNAi target for antiviral therapy. These findings provide useful information for the development of RNAi-based prophylaxis and therapy for human influenza virus infection.

## Introduction

Influenza A virus (IAV) remains a scourge on human health [1], [2], [3]. Its antigen drifts and shifts are an ever-changing challenge for available vaccines [4], [5]. The appearance of drug resistance is the main hurdle for the development of antiviral drugs [6], [7], [8], [9]. Given the limitations of current anti-influenza A virus strategies, the need for novel strategies for prevention and treatment of IAV is evident [10]. In this regard, RNA interfering (RNAi) technology holds great promise to inhibit the replication of IAV, including H5N1 virus.

RNAi is a form of posttranscriptional gene silencing mediated by short double-stranded RNA, known as small interfering RNA (siRNA) [11], [12]. In this process, the cellular complex Dicer cleaves a double-stranded RNA (dsRNA) molecule to yield double-stranded duplexes 21–25 nucleotides in length. These siRNAs then guide the RNAi induced silencing complex (RISC) to cleave target mRNAs that share sequence identity with the siRNA [13], [14], [15]. Since it was first demonstrated that adding exogenous, synthetic siRNA molecules to mammalian cells can induce RNAi, there have been rapidly expanding efforts to develop RNAi therapies that induce the degradation of target messenger RNA (mRNA) involved in genetically inherited diseases or acquired disorders [16], [17], [18], [19], [20], [21], [22].

IAV is an enveloped, negative-stranded RNA virus. The unique property of single-stranded RNA virus itself makes RNAi an attractive approach for development of anti-avian influenza therapeutics. The single-stranded viral genome, consisting of 8 segments contained at least 10 open reading frames (ORFs), serves as template for both viral genome replication and subgenomic mRNA synthesis. It has been reported that siRNAs respectively targeting to the viral genes of polymerase 1 (PB1), polymerase 2 (PB2), polymerase A (PA), nucleocapsid protein (NP), non-structure proteins (NS1 and NS2), matrix proteins (M1 and M2), especially those specific for NP, PA and PB1, can potently inhibit replication of influenza A viruses [16], [23], [24], [25], [26]. However, it has been reported that HIV and HCV may develop siRNA-resistant mutations quickly [17], [27], [28], and therefore abrogated the further RNAi treatment. Thus, the evaluation of long term inhibition efficiency of designed siRNAs and screening of the emergence of siRNA resistance mutants are also an important research target.

In the present study, we identified an effective siRNA targeting M2 gene (siM2), a highly conserved gene in IAV, as compared to a reported effective siRNA targeting NP gene (siNP). We further established cell lines which stably expressing the shRNAs by transducing lentiviral-shRNA vectors to Madin-Darby cannie kidney (MDCK) cells. Using these two cell lines, we evaluated long term antiviral effects of these siRNAs against IAV subtypes H1N1 and H5N1 and further screened the potential siRNA-resistant viral mutations. Our results showed that rationally designed siM2 conferred long term effective inhibition for IAV replication. It was further demonstrated that no siRNA-resistant viral mutation appeared in siM2 targeting sequence even after the virus was cultured in the shRNA expressing stable cell line for 40 passages.

## Results

### Screening Effective siRNAs Targeting M2 Gene

**Figure 1:**
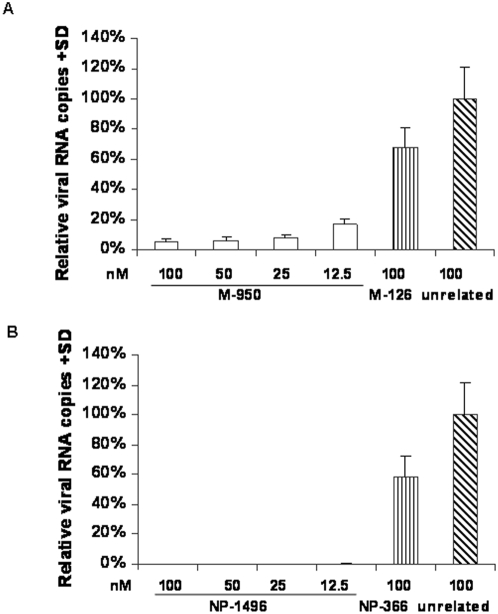
Effects of chemically synthesized siRNAs on influenza virus production. (A) siRNA M-950 is effective for influenza virus inhibition. (B) siRNA NP-1496 can inhibit influenza virus replication at indicated siRNA concentration. An unrelated siRNA targeting GFP was used as negative control. MDCK cells were transfected by chemically synthesized siRNAs, and infected with H1N1 virus at a moi of 0.005 in 8 hrs after transfection. Viral culture supernatants were collected at 48 hrs post infection. The viral load was detected by Q RT-PCR and expressed by relative viral RNA copies. The data were from three replicates of the experiments and presented as the mean value+SD.

Two siRNAs targeting the M2 gene were rationally designed by siRNA target designer (the sequences of siRNAs are shown in the supporting information Table S1) and their effect in inhibiting the virus replication was assessed in MDCK cells. Two siRNAs targeting the NP gene were included in the experiments as controls. The results showed the siRNA M-950 exhibited a good inhibition effect with dose dependent manner, while another siRNA M-126 just slightly inhibited virus replication even at a concentration of 100 nM (Fig. 1A). Fig. 1B showed that the siRNA NP-1496 could inhibit influenza virus replication, while siRNA NP-336 had no inhibition effect, which is consistent with the previous report [25].

### The siM2 Exhibited Higher Inhibitory Effect of H1N1 Virus than siNP in Stable Cell Lines

**Figure 2:**
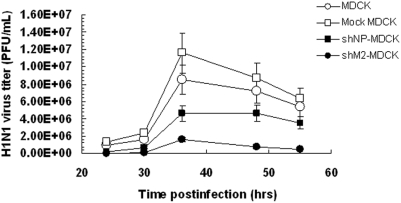
Inhibition of influenza H1N1 virus production on stable cell lines. Stable cell lines were infected by H1N1 virus at a moi of 0.005. Virus titer was measured at the indicated time points after infection. Data were from three replicates of the experiments.

Based on the above results, the lentiviruses expressing the shRNAs M2-950 or NP-1496 were constructed and transduced into MDCK cells to establish two stable cell lines, shM2-MDCK and shNP-MDCK. MDCK cells and the MDCK cells transduced by blank lentivirus (Mock MDCK) were used as controls. The cell lines were infected with H1N1 virus at a moi of 0.005 and culture supernatants were harvested at indicated time-points to determine the virus titer by plaque assay. As shown in Fig. 2, virus replication kinetics of Mock MDCK is similar with that of MDCK, indicating that lentivirus integration didn't influence virus replication. Virus titers in shNP- and shM2-MDCK cell cultures were 2 to 10 folds lower than the controls MDCK and Mock MDCK cultures, suggesting that virus replication had been suppressed by the expressed shRNAs in both shM2-MDCK and shNP-MDCK cells. Notably, siM2 exhibited a better inhibition effect, showing about 2-fold lower viral titer than siNP, although the expression levels of siM2 and siNP were similar (ΔCt siM2 = 6.68, siNP = 6.95).

### The siM2 Abolished not only M2 mRNA but also siNP mRNA Accumulation in the Stable Cell Lines

**Figure 3:**
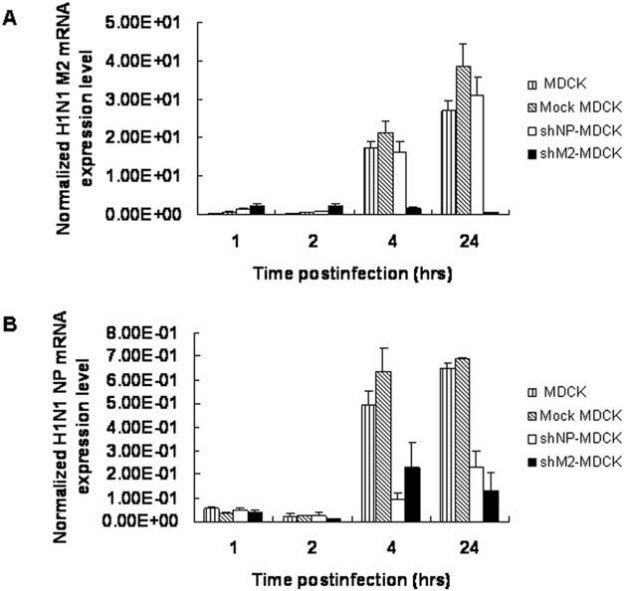
Viral mRNA levels are decreased in stable shRNA-expressing cell lines infected by H1N1. (A) siM2 inhibit M2 specific mRNA expression level (B)siNP inhibit NP specific mRNA expression level. Stable cell lines were infected by H1N1 at a moi of 0.005, mRNA was isolated at indicated time after infection. M2 or NP specific mRNA expression level was detected by Q RT-PCR and the results were normalized by copy number of β-actin. The experiments were repeated three times and the data were presented as the mean values+SD.

We also measured the accumulation of mRNA for NP and M2 gene in infected MDCK, Mock MDCK, shM2-MDCK and shNP-MDCK cells. The mRNAs were extracted from the cells harvested at 1, 2, 4 and 24 hrs post-infection and tested by real-time RT-PCR. The mRNA expression level is normalized by copy number of β-Actin. The M2 mRNA level in shM2-MDCK cells harvested at 4 hrs and 24 hrs post-infection was significantly lower than those in MDCK, Mock MDCK and shNP-MDCK cells (Fig. 3A). Similarly, the NP mRNA level in shNP-MDCK cells collected at 4 hrs and 24 hrs after the viral infection was significantly suppressed as compared to those in MDCK and Mock MDCK cells (Fig. 3B). Interestingly, siM2 could also inhibit the accumulation of NP mRNA (Fig. 3B), suggesting that the siM2 might have a broad inhibition effect.

### NP Protein Expression was Suppressed in Virus Infected shM2-MDCK Cells

**Figure 4:**
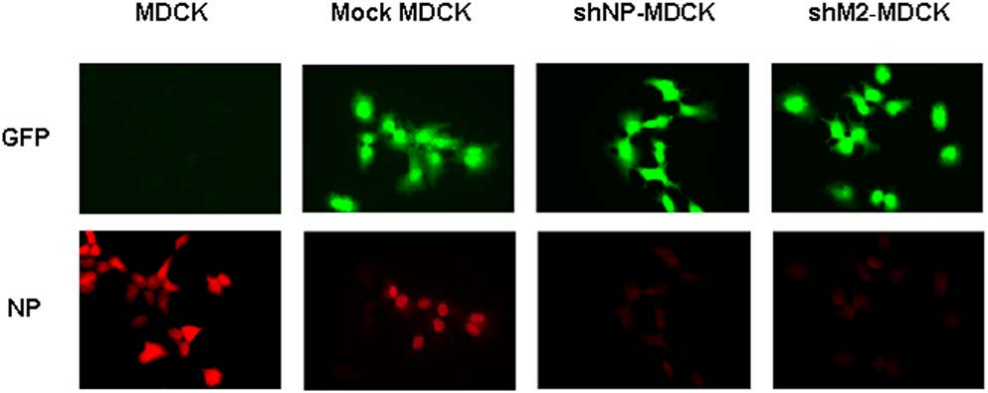
Viral protein levels in stable shRNA-expressing cell lines infected by H1N1 virus. The indicated cell lines were infected with H1N1 virus for 6 hrs and stained with anti-NP antibody. Green represents signal from EGFP in the lentiviral vectors, while red (texas red) represents staining with anti-NP antibody. Images were taken by using a fluorescence microscope under a 400 magnification. All the pictures were captured under the same exposure time and gain.

To further confirm whether the suppression of NP mRNA in shM2-MDCK cells indeed affect NP protein expression, the NP protein level was tested by an indirect immunofluorescence assay. As shown in Fig. 4, EGFP fluorescence, an indicator of shRNA expression, was detected in Mock MDCK, shNP-MDCK and shM2-MDCK but not in MDCK cells, while NP protein was detected in MDCK and Mock MDCK cells but not in shNP-MDCK and shM2-MDCK cells. The results were consistent with above viral mRNA results, indicating that siM2 indeed suppressed the NP protein expression.

### siM2 Provided More Potent anti-H5N1 Viral Effect than siNP in Stable Cell Lines

**Figure 5:**
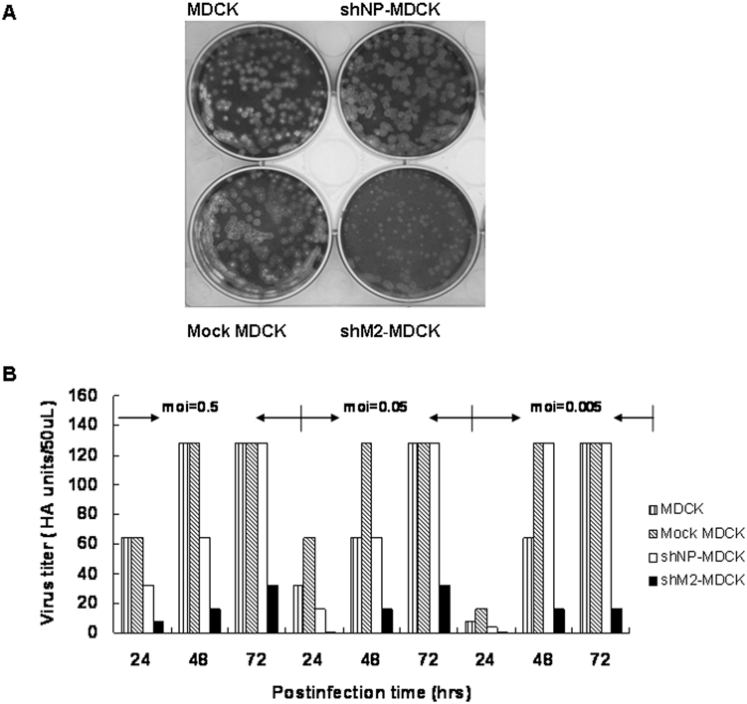
Inhibition of influenza H5N1 virus production on stable shRNA expressing cell lines. (A) Inhibitory effects of siM2 and siNP were detected by plaque reduction assay after the stable cell lines were infected with 0.05 moi of H5N1 virus for 72 hours. (B) The stable cell lines were infected with indicated doses of H5N1 virus. Virus titers were measured at the indicated time points after infection. The data were presented as the mean values of two experiments.

We further tested whether siM2 could also inhibit the replication of a highly pathogenic H5N1 avian influenza virus. As shown in Fig. 5A, although numbers of plaques were similar in different MDCK cell lines, smaller size of plaques were only found in shM2-MDCK cells, suggesting that siM2 inhibited replication of H5N1 virus. The cell lines were also infected with different amounts of H5N1 virus and culture supernatants were collected at different time points to determine the virus titers by HA assay. The virus replication was significantly inhibited in shM2-MDCK cells at all time-points, but shNP-MDCK just offered a minor inhibition effect at early stage of the virus infection (Fig. 5B). These results further confirmed that siM2 could provide a more potent protection than siNP against H5N1 infection.

### siM2 Resistant Virus Mutant was not Observed Even after 40 Passages

**Figure 6:**
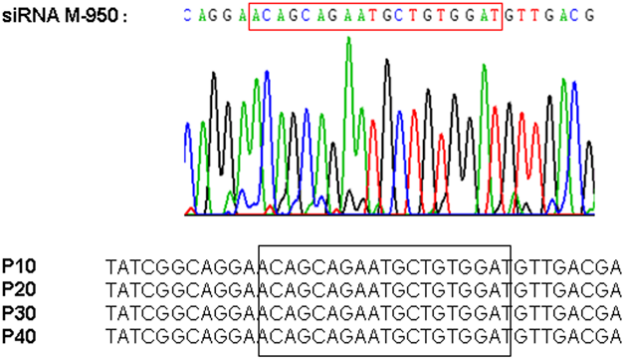
Screening for siRNA resistant mutants in shM2-MDCK stable cell line. Influenza A virus was cultured in shM2-MDCK for 40 passages and sequencing of siM2 targeted region in 10 plaque purified virus isolates revealed the parental sequence. P10, P20, P30 and P40 are representative passages of viruses.

To test if siM2 siRNA-resistant virus mutant would quickly appeared when cultured in shM2-MDCK cells, H5N1 virus was continually cultured in shM2-MDCK cells for 40 passages. Every 10 passages, the culture supernatant was collected and tested by plaque reduction assay. No obvious larger size of plaque was found. Ten plaques with relative larger size were picked to further identify potential mutation in the siRNA targeting region by sequencing. The results showed that no mutation appeared in the siM2 targeting region even after 40 passages of the cultures (Fig. 6).

## Discussion

The principal finding of this study is that rationally designed siRNA targeting influenza M2 gene (M-950) conferred effective long term inhibition against influenza A virus replication. Such high suppressive effect is not only against H1N1 influenza A virus but also against a highly pathogenic H5N1 subtype. In the previous related studies, Ge and his co-workers [25] screened siRNAs targeting to 6 conserved genes of influenza A virus and showed that NP-1496 was the best since it can confer a more than 200-folds inhibition of H1N1 virus. Li et al [29] and Tomkines et al [23] further confirmed that NP-1496 provided high anti-H5N1 effect. We therefore included NP-1496 as a positive control in this study. Our results showed that siRNA M-950 exhibited similar (Fig. 1) or even slight higher (Fig. 2) inhibitory effect against IAV replication as compared to that of NP-1496. A recent report by Zhou et al [30] also showed that several siRNAs targeting NP and M genes exhibited effective inhibition against influenza A virus replication in cultured MDCK cells and in animal models. However, sequences of their reported siRNAs targeting M2 gene are completely different from the siRNA M2-950. Furthermore, chemically synthesized siRNAs or plasmid based shRNAs were always delivered by transfection in previous related studies, whereas we used a lentivirus system to deliver selected shRNAs. Although the integration property of lentivirus has abrogated it to be used in human, it is helpful for our study purpose to successfully establish stable cell lines persistently expressing siRNAs.

In this study we found that siM2 not only decreased the level of M2 mRNA but also the level of NP mRNA, suggesting that siM2 has a broad inhibition manner in the process of influenza virus replication. Ge et al have reported a similar broad inhibition of siRNAs [25]. In their study, NP-1496 and PA-2087 provided a broad inhibition to H1N1 influenza virus, which not only abolished the accumulations of specific NP or PA mRNAs but also inhibited the accumulations of mRNAs for M, NS1, PB1, PB2 and PA or NP genes. A possible explanation is that some double stranded siRNAs may result in IFN responses or activate a RNA degradation pathway, e.g. Phosphorylated protein Kinase R (PKR) [9], [31], [32]. However, the mechanisms of this broad inhibition of some siRNAs are still not very clear yet. From the standpoint of viral target choice in RNAi based antiviral therapy, NP protein is required for elongation and antitermination of nascent cRNA and vRNA transcripts [33], [34]. Without newly synthesized NP, further viral transcription and replication are blocked. While, M2 plays a critical role in the assembly of infectious virus particles. Thus, the potent antiviral effect of siM2 may be attributed to its broad inhibitory effect.

Depending on the stringency of siRNA-target base pairing, siRNA treatment may cause selection of siRNA-resistant viruses, and this has been shown with HIV and HCV [17], [27], [28], and therefore abrogated the further medication or treatments. Using lentiviral delivery system, we established stable cell lines persistently expressing shRNA, which provided a more convenient experimental approach to study long term inhibition effect of siRNAs and screen for siRNA resistant virus mutants in quasispecies *in vitro*. Our results showed that H5N1 virus cultured in shM2-MDCK were equally susceptible to siM2 as the original virus even after 40 passages. Moreover, sequencing of siM2 targeted region in 10 such independent plaque purified virus isolates revealed sequence identical to the parental one. The current data have shown no insertion, deletion and nucleotide substitution in the siRNA target sequence, therefore demonstrated siM2 possessed good long term inhibition effect for influenza virus replication without the problem of siRNA resistant mutants.

Taken together, all the findings about effective RNAi target, lentiviral vector delivery and the establishment of stable shRNA expressing cell lines in our study provide rational information for the development of siRNAs as prophylaxis and therapy for influenza virus infection in humans.

## Materials and Methods

### Cell lines and viruses

MDCK and Human embryonic kidney 293T cells were respectively maintained in MEM and DMEM (Invitrogene, USA) supplemented with 10% heat-inactivated fetal bovine serum (FBS) and antibiotics (100 U penicillin G/mL and 100 ug streptomycin/mL). Influenza virus strains A/New Caledonia/20/1999 (H1N1) and A/Hong Kong/486/97 (H5N1) used in these experiments were prepared in MDCK cells and virus titers were determined by TCID_50_. All experiments with H5N1 virus were performed in BSL-3 laboratory.

### Preparation and transfection of siRNAs

The siRNAs targeting M or NP gene of influenza A virus were designed by siRNA target designer version 1.51 from Promega (http://www.promega.com/siRNADesigner/program/). The duplexes of designed and previously reported siRNAs were synthesized by Invitrogene (USA) (the sequences were shown in the supporting information Table S1). The siRNAs were reverse transfected to MDCK cells using Lipofectamine™ RNAiMAX (Invitrogene, USA) as described in company's instruction. After incubated the cells for 16∼18 hrs, the cells were infected with the viruses and followed by detection of viral replication. 24 hours after infection, RNA were extracted from the cells and followed by real time RT-PCR to detect the relative quantities of replicated viral RNA.

### Construction of lentiviral vectors

The H1-promoter-driven shRNA cassettes were constructed by annealing two primers containing the 19-nt sense and reverse complementary targeting sequences with a 9-nucleotide loop -TTCAAGAGA- and flanking Mlu1 and Cla1 cloning sites (the sequences of shRNA were shown in the supporting information Table S1), and then cloned into the 3′-end of the H1 promoter in the LVTHM plasmid [35], [36]. The sequences of the insertions were confirmed by DNA sequencing.

### Generation of recombinant lentivirus

Lentiviral vectors with shRNA expression cassette were produced by calcium phosphate-mediated, three-plasmid transfection of 293T cells [37]. Briefly, 293T cells (2.5×10^6^ cells in a 75T flask) were transfected with 20 µg LVTHM or LVTHM-shRNA, 15 µg psAX2 and 6 µg pMD.2G and cultured in DMEM supplemented with 10% FBS and antibiotics. Virus supernatants were collected on day 3 post-transfection, filtered through a 0.45 µm pore-size filter, ultracentrifuged at 40,000 *g* for 3 hrs at 4°C and resuspended in PBS. Virus stocks were titrated by infecting Hela cells with virus dilutions in DMEM and 8 µg polybrene (hexadimethrine bromide, Sigma) ml^−1^ and analyzed for EGFP expression with a flow cytometer (BD Bioscience Immunocytometry Systems, USA). Data was processed with Cellquest software. Titers of the virus stocks were routinely 10^7^∼10^8^ transduction units (TU) ml^−1^.

### Establishment of shRNA expressing stable cell lines

Lentiviral stocks were used to transduce MDCK cells. After 3 days of transduction, the medium containing the lentivirus was replaced with complete culture medium. The transduced MDCK was sub-cultured every 3–4 days for 4 weeks to get stable viral genome integration. Sorting of live GFP positive cells was performed using a FACStar+ instrument (Becton Dickinson, USA) [38]. Established stable expressing cell lines were named as follows: Mock MDCK, MDCK cells transduced by lentivirus without shRNA insertion; shNP-MDCK, MDCK cells transduced by lentivirus with shNP insertion (NP-1496); shM2-MDCK, MDCK cells transduced by lentivirus with shM2 insertion (M-950). The amounts of shRNAs expression in established lines of shM2-MDCK and shNP-MDCK were quantified by Q RT-PCR using forward primers siM2- 5′-ACA GCA GAA TGC TGT GGA T-3′ and siNP- 5′-GGA TCT TAT TTC TTC GGA G-3′, and the reverse primer provided by Ncode™ miRNA Q-RT-PCR kit (Invitrogene, USA) and further calculated with normalized Ct values: ΔCt (Mock MDCK−shM2-MDCK or shNP-MDCK).

### Influenza virus infection and viral load detection

MDCK and the stable shRNA expressing cell lines in 24-well plates were infected with viruses at moi of 0.005∼0.5 (2 µg/mL trypsin was used in the infection process of H1N1). After incubation for 1 hr, the infected medium was removed and MEM without FBS was added. Cell supernatants were collected at different time points. The viral load was detected by hemagglutination (HA) and/or plaque assays as described previously [39]. Briefly, the HA assay was carried out in U-bottom 96 well plates. Serial 2-fold dilutions of virus samples were mixed with an equal volume of a 0.5% suspension of turkey erythrocytes (Lampire Biologic Laboratories, Pipersville, USA) and incubated at room temperature (RT) for 45 mins. Wells containing an adherent, homogeneous layer of erythrocytes were scored as positive. For plaque assay, serial 10-fold dilutions of virus sample were added into a monolayer of MDCK cells. After 1 hr incubation, the virus was removed and the cultures were overlaid with 1% semi solid agar-MEM. Three days after infection, plaques were visualized by staining of crystal violent.

### Real-time RT-PCR

Real-time RT-PCR was carried out as described previously [39]. Briefly, H1N1 or H5N1 virus infected MDCK, Mock MDCK, shNP-MDCK and shM2-MDCK were harvest at 1, 2, 4 and 24 hr after infection. Total RNA was extracted from the infected cell samples using RNeasy RNA isolation Kit (Qiagen, Germany) and reverse transcribed using Superscript II Reverse Transcriptase and Oligo dT primer (Invitrogene, USA), according to the manufacturer's protocol. Viral mRNA copies were measured by SYBR green M×3000 Real-Time PCR System (Stratagene, USA), using primers NP-Forward: 5′-GAC CAG GAG TGG AGG AAA CA-3′, NP-Reverse: 5′-CGG CCA TAA TGG TCA CTC TT-3′; M2-Forward: 5′-CGT CGC TTT AAA TAC GGT TTG-3′, M2-Reverse: 5′- CGT CAA CAT CCA CAG CAT TC-3′ β-Actin-Forward: 5′- CGT ACC ACT GGC ATC GTG AT-5′, β-Actin-Reverse: 5′-GTG TTG GCG TAC AGG TCT TTG-3′. The reactions were performed at 95°C 10 mins, 40 cycles of 95°C 1 min, 60°C 1 min, 72°C 1 min, followed by melting curve analysis according to instrument documentation (Stratagene M×3000). All reactions were done in triplicates and the results were normalized by β-action.

### Indirect Immunofluorescence Assay

Indirect immunofluorescence assay was performed as described previously [40], [41] with some modification. MDCK, Mock MDCK, shNP-MDCK and shM2-MDCK cells grew on micro cover glasses (Thomas, USA) were infected with 1 moi of H1N1 virus for 6 hrs, After washed with PBS, the cells were fixed in 4% paraformaldehyde for 15 mins at RT and then permeabilized in 0.1% Triton X-100 for 3 mins at RT. After washed with PBS again, the cells were incubated with 1∶50 diluted mouse anti-NP antibody (Abcam, UK) for 30 mins in dark at RT. The cells were washed three times in PBS with 1% FCS and incubated with 1∶500 diluted Texas red-conjugated anti-mouse lgG (Abcam, UK) for 30 mins in the dark at RT. The cells were washed and mounted. Slides were viewed under an Olympus fluorescence microscope (Olympus, Germany).

### Screening siRNA resistant mutants on shM2-MDCK

The screening of potential siRNA resistant mutants were performed in our established stable shRNA-expressing cell lines according to previously described protocols [42] with some modification. Briefly, the shM2-MDCK cells in a T25 cm^2^ flask were infected with H5N1 virus. After cultured for 2 days, the supernatants were harvested. Part of the supernatants was inoculated to shM2-MDCK for next passage of the virus culture, another part was subjected for plaque assay to determine if potential siRNA-resistant virus appeared. Every 10 passages, ten bigger size of plaques in the plaque assay were picked for sequencing to detect any mutation in the siRNA targeting region using a pair of primers: forward, 5′-AAG GCA GAT GGT GCA GGC AAT-3′ and reverse, 5′-TAC TCC AGC TCT ATG CTG ACA-3′.

## Supporting Information

Table S1 (0.03 MB DOC)

## References

[1] Kandun IN, Wibisono H, Sedyaningsih ER, Yusharmen, Hadisoedarsuno W (2006). Three Indonesian clusters of H5N1 virus infection in 2005.. N Engl J Med.

[2] Ungchusak K, Auewarakul P, Dowell SF, Kitphati R, Auwanit W (2005). Probable person-to-person transmission of avian influenza A (H5N1).. N Engl J Med.

[3] Wang H, Feng Z, Shu Y, Yu H, Zhou L (2008). Probable limited person-to-person transmission of highly pathogenic avian influenza A (H5N1) virus in China.. Lancet.

[4] Tran TH, Nguyen TL, Nguyen TD, Luong TS, Pham PM (2004). Avian influenza A (H5N1) in 10 patients in Vietnam.. N Engl J Med.

[5] Yen HL, Monto AS, Webster RG, Govorkova EA (2005). Virulence may determine the necessary duration and dosage of oseltamivir treatment for highly pathogenic A/Vietnam/1203/04 influenza virus in mice.. J Infect Dis.

[6] Degelau J, Somani SK, Cooper SL, Guay DR, Crossley KB (1992). Amantadine-resistant influenza A in a nursing facility.. Arch Intern Med.

[7] Gubareva LV, Robinson MJ, Bethell RC, Webster RG (1997). Catalytic and framework mutations in the neuraminidase active site of influenza viruses that are resistant to 4-guanidino-Neu5Ac2en.. J Virol.

[8] Houck P, Hemphill M, LaCroix S, Hirsh D, Cox N (1995). Amantadine-resistant influenza A in nursing homes. Identification of a resistant virus prior to drug use.. Arch Intern Med.

[9] Kumar M, Carmichael GG (1998). Antisense RNA: function and fate of duplex RNA in cells of higher eukaryotes.. Microbiol Mol Biol Rev.

[10] Beigel JH, Farrar J, Han AM, Hayden FG, Hyer R (2005). Avian influenza A (H5N1) infection in humans.. N Engl J Med.

[11] Bernstein E, Caudy AA, Hammond SM, Hannon GJ (2001). Role for a bidentate ribonuclease in the initiation step of RNA interference.. Nature.

[12] Hammond SM, Bernstein E, Beach D, Hannon GJ (2000). An RNA-directed nuclease mediates post-transcriptional gene silencing in Drosophila cells.. Nature.

[13] Engelke DR, Rossi JJ (2005). RNA interference.

[14] Appasani K (2004). RNA interference technology : from basic science to drug development.

[15] Doi N, Zenno S, Ueda R, Ohki-Hamazaki H, Ui-Tei K (2003). Short-interfering-RNA-mediated gene silencing in mammalian cells requires Dicer and eIF2C translation initiation factors.. Curr Biol.

[16] Li BJ, Tang Q, Cheng D, Qin C, Xie FY (2005). Using siRNA in prophylactic and therapeutic regimens against SARS coronavirus in Rhesus macaque.. Nat Med.

[17] Konishi M, Wu CH, Kaito M, Hayashi K, Watanabe S (2006). siRNA-resistance in treated HCV replicon cells is correlated with the development of specific HCV mutations.. J Viral Hepat.

[18] Moore MD, McGarvey MJ, Russell RA, Cullen BR, McClure MO (2005). Stable inhibition of hepatitis B virus proteins by small interfering RNA expressed from viral vectors.. J Gene Med.

[19] Scherr M, Battmer K, Winkler T, Heidenreich O, Ganser A (2003). Specific inhibition of bcr-abl gene expression by small interfering RNA.. Blood.

[20] Wohlbold L, van der Kuip H, Miething C, Vornlocher HP, Knabbe C (2003). Inhibition of bcr-abl gene expression by small interfering RNA sensitizes for imatinib mesylate (STI571).. Blood.

[21] Song E, Lee SK, Wang J, Ince N, Ouyang N (2003). RNA interference targeting Fas protects mice from fulminant hepatitis.. Nat Med.

[22] Zender L, Hutker S, Liedtke C, Tillmann HL, Zender S (2003). Caspase 8 small interfering RNA prevents acute liver failure in mice.. Proc Natl Acad Sci U S A.

[23] Tompkins SM, Lo CY, Tumpey TM, Epstein SL (2004). Protection against lethal influenza virus challenge by RNA interference in vivo.. Proc Natl Acad Sci U S A.

[24] Hui EK, Yap EM, An DS, Chen IS, Nayak DP (2004). Inhibition of influenza virus matrix (M1) protein expression and virus replication by U6 promoter-driven and lentivirus-mediated delivery of siRNA.. J Gen Virol.

[25] Ge Q, McManus MT, Nguyen T, Shen CH, Sharp PA (2003). RNA interference of influenza virus production by directly targeting mRNA for degradation and indirectly inhibiting all viral RNA transcription.. Proc Natl Acad Sci U S A.

[26] Ge Q, Filip L, Bai A, Nguyen T, Eisen HN (2004). Inhibition of influenza virus production in virus-infected mice by RNA interference.. Proc Natl Acad Sci U S A.

[27] Das AT, Brummelkamp TR, Westerhout EM, Vink M, Madiredjo M (2004). Human immunodeficiency virus type 1 escapes from RNA interference-mediated inhibition.. J Virol.

[28] Westerhout EM, Ooms M, Vink M, Das AT, Berkhout B (2005). HIV-1 can escape from RNA interference by evolving an alternative structure in its RNA genome.. Nucleic Acids Res.

[29] Li YC, Kong LH, Cheng BZ, Li KS (2005). Construction of influenza virus siRNA expression vectors and their inhibitory effects on multiplication of influenza virus.. Avian Dis.

[30] Zhou H, Jin M, Yu Z, Xu X, Peng Y (2007). Effective small interfering RNAs targeting matrix and nucleocapsid protein gene inhibit influenza A virus replication in cells and mice.. Antiviral Res.

[31] Garcia-Sastre A (2002). Mechanisms of inhibition of the host interferon alpha/beta-mediated antiviral responses by viruses.. Microbes Infect.

[32] Katze MG, He Y, Gale M (2002). Viruses and interferon: a fight for supremacy.. Nat Rev Immunol.

[33] Beaton AR, Krug RM (1986). Transcription antitermination during influenza viral template RNA synthesis requires the nucleocapsid protein and the absence of a 5′ capped end.. Proc Natl Acad Sci U S A.

[34] Shapiro GI, Krug RM (1988). Influenza virus RNA replication in vitro: synthesis of viral template RNAs and virion RNAs in the absence of an added primer.. J Virol.

[35] Arrighi JF, Pion M, Wiznerowicz M, Geijtenbeek TB, Garcia E (2004). Lentivirus-mediated RNA interference of DC-SIGN expression inhibits human immunodeficiency virus transmission from dendritic cells to T cells.. J Virol.

[36] Szulc J, Wiznerowicz M, Sauvain MO, Trono D, Aebischer P (2006). A versatile tool for conditional gene expression and knockdown.. Nat Methods.

[37] An DS, Xie Y, Mao SH, Morizono K, Kung SK (2003). Efficient lentiviral vectors for short hairpin RNA delivery into human cells.. Hum Gene Ther.

[38] Fish RJ, Kruithof EK (2004). Short-term cytotoxic effects and long-term instability of RNAi delivered using lentiviral vectors.. BMC Mol Biol.

[39] Zheng BJ, Chan KW, Lin YP, Zhao GY, Chan C (2008). Delayed antiviral plus immunomodulator treatment still reduces mortality in mice infected by high inoculum of influenza A/H5N1 virus.. Proc Natl Acad Sci U S A.

[40] Zheng B, Graham FL, Johnson DC, Hanke T, McDermott MR (1993). Immunogenicity in mice of tandem repeats of an epitope from herpes simplex gD protein when expressed by recombinant adenovirus vectors.. Vaccine.

[41] Zheng BJ, Chan KW, Im S, Chua D, Sham JS (2001). Anti-tumor effects of human peripheral gammadelta T cells in a mouse tumor model.. Int J Cancer.

[42] Kinchington D, Schinazi RF (2000). Antiviral methods and protocols.

