# Supplementary material for: Small Interfering RNA Targeting M2 Gene Induces Effective and Long Term Inhibition of Influenza A Virus Replication
Source: PLoS One. 2009 May 22;4(5):e5671. doi: 10.1371/journal.pone.0005671 (PMC2682565; doi:10.1371/journal.pone.0005671)
Supplement: Table S1 — (0.03 MB DOC) [file pone.0005671.s001.doc]

Supporting information

**Table S1** Sequences of siRNAs and shRNAs

| **Names** | **Sequences** |
| --- | --- |
| siM2 (M-126) | 5’-GGA AGA ACA CAG AUC UUG A-3’  5’-UCA AGA UCU GUG UUC UUC C-3’ |
| siM2 (M-950) | 5’-ACA GCA GAA UGC UGU GGA U-3’  5’-AUC CAC AGC AUU CUG CUG U-3’ |
| siNP (NP-366) | 5’-GCU GAU UCU GUA UGA CAA A-3’  5’-UUU GUC AUA CAG AAU CAG C-3 |
| siNP (NP-1496)* | 5’-GGA UCU UAU UUC UUC GGA G-3’  5’-CUC CGA AGA AAU AAG AUC C-3’ |
| Unrelated siRNA:  siGFP (GFP-949) | 5’-GGC UAC GUC CAG GAG CGC AUU-3’  5’-UUC CGA UGC AGG UCC UCG CGU-3’ |
| shM2 (M-950) | 5’-**CGC GT**C CCC ACA GCA GAA UGC UGU GGA UTT CAA GAG AAC AGC AGA AUG CUG UGG AUT TTT TGG AA**A T**-3’  3’-**A**GG GGU GUC GUC UUA CGA CAC CUA AAG TTC TCT UGU CGU CUU ACG ACA CCU AAA AAA CCT T**TA GC**-5’ |
| shNP (NP-1496) | 5’-**CGC GT**C CCC GGA UCU UAU UUC UUC GGA GTT CAA GAG AGG AUC UUA UUU CUU CGG AGT TTT TGG AA**A T**-3’  3’-**A**GG GGC CUA GAA UAA AGA AGC CUC AAG TTC TCT CCU AGA AUA AAG AAG CCU CAA AAA CCT T**TA GC**-5’ |

* This siRNA has been reported [25] and included in this study as positive control.

For shRNA, Mlu1 and Cla1 cloning sites and the 19-nt sense and reverse complementary targeting sequences are indicated by bold and underline respectively.
